# Randomized Controlled Trial of Levamisole Hydrochloride as Adjunctive Therapy in Severe Falciparum Malaria With High Parasitemia

**DOI:** 10.1093/infdis/jit410

**Published:** 2013-08-13

**Authors:** Richard J. Maude, Kamolrat Silamut, Katherine Plewes, Prakaykaew Charunwatthana, May Ho, M. Abul Faiz, Ridwanur Rahman, Md Amir Hossain, Mahtab U. Hassan, Emran Bin Yunus, Gofranul Hoque, Faridul Islam, Aniruddha Ghose, Josh Hanson, Joel Schlatter, Rachel Lacey, Alison Eastaugh, Joel Tarning, Sue J. Lee, Nicholas J. White, Kesinee Chotivanich, Nicholas P. J. Day, Arjen M. Dondorp

**Affiliations:** 1 Mahidol-Oxford Tropical Medicine Research Unit, Faculty of Tropical Medicine, Mahidol University, Bangkok, Thailand; 2 Centre for Tropical Medicine, Nuffield Department of Clinical Medicine, University of Oxford, United Kingdom; 3 Department of Microbiology and Infectious Disease, University of Calgary, Alberta, Canada; 4 Centre for Specialized Care and Research, Chittagong; 5 Dev Care Foundation, Dhaka; 6 Shaheed Shwarwardhy Medical College, Dhaka; 7 Department of Medicine, Chittagong Medical College Hospital, Chittagong, Bangladesh; 8 Department of Pharmacy and Toxicology, University Hospital of Jean Verdier, Bondy, France; 9 Worcestershire Royal Hospital, Worcester, United Kingdom

**Keywords:** malaria, severe, falciparum, sequestration, artesunate, levamisole

## Abstract

***Background.*** Cytoadherence and sequestration of erythrocytes containing mature stages of *Plasmodium falciparum* are central to the pathogenesis of severe malaria. The oral anthelminthic drug levamisole inhibits cytoadherence in vitro and reduces sequestration of late-stage parasites in uncomplicated falciparum malaria treated with quinine.

***Methods.*** Fifty-six adult patients with severe malaria and high parasitemia admitted to a referral hospital in Bangladesh were randomized to receive a single dose of levamisole hydrochloride (150 mg) or no adjuvant to antimalarial treatment with intravenous artesunate.

***Results.*** Circulating late-stage parasites measured as the median area under the parasite clearance curves were 2150 (interquartile range [IQR], 0–28 025) parasites/µL × hour in patients treated with levamisole and 5489 (IQR, 192–25 848) parasites/µL × hour in controls (*P* = .25). The “sequestration ratios” at 6 and 12 hours for all parasite stages and changes in microvascular blood flow did not differ between treatment groups (all *P* > .40). The median time to normalization of plasma lactate (<2 mmol/L) was 24 (IQR, 12–30) hours with levamisole vs 28 (IQR, 12–36) hours without levamisole (*P* = .15).

***Conclusions.*** There was no benefit of a single-dose of levamisole hydrochloride as adjuvant to intravenous artesunate in the treatment of adults with severe falciparum malaria. Rapid parasite killing by intravenous artesunate might obscure the effects of levamisole.

Severe malaria in adults has a high mortality rate between 15% and 30%, and no adjunctive treatment has been able to reduce this unacceptably high figure [1]. Pivotal in the pathogenesis of cerebral and severe *Plasmodium falciparum* malaria is sequestration of erythrocytes containing mature stages of the parasite, which is augmented by endothelial cell activation and dysfunction, culminating in microvascular obstruction [2, 3]. A cheap and safe drug targeting cytoadherence and thus sequestration in vital organs would be a promising candidate for adjunctive treatment in severe falciparum malaria.

Cytoadherence is mediated by the parasite protein *P. falciparum* erythrocyte membrane protein 1 (*Pf*EMP1) [4], and endothelial receptor molecules, including CD36 [5, 6] intercellular adhesion molecule 1 (ICAM-1) [7], E-selectin, and vascular cell adhesion molecule 1 (VCAM-1) [8]. Studies under physiological flow conditions have shown that the different adhesion molecules interact synergistically. The infected red blood cells tether and roll on endothelial receptors with a low affinity (ICAM-1, VCAM-1, and P-selectin), facilitating the subsequent more-definite adhesion to CD36 [9–12]. Peptide mapping studies have revealed that the critical region on *Pf*EMP1 involved in binding to CD36 is localized to a 179–amino acid sequence within the cysteine-rich interdomain region 1 (CIDR-1). A recombinant 179–amino acid peptide expressed in yeast (PpMC-179) represents this minimal CD36-binding domain and is able to reduce cytoadherence of *P. falciparum*–infected red blood cells (IRBCs) by >80% in a human/severe combined immunodeficiency (SCID) mouse chimeric model [13]. It was shown more recently that binding of PpMC-179 to CD36 on human dermal microvascular endothelial cells activates a physically closely associated Src-family kinase [14, 15]. This activation results in an increased affinity of CD36 for IRBCs through activation of an ecto-alkaline phosphatase (ecto-ALP), causing dephosphorylation of the extracellular domain of CD36 [16]. Selective inhibition of the Src-family kinase by the pyrazolopyrimidine PP1 inhibits adhesion of IRBC by >70% in a flow-chamber assay and in a human/SCID mouse chimeric model.

Levamisole hydrochloride is an old and cheap drug, primarily used to treat intestinal helminths. It is a specific alkaline-phosphatase inhibitor, and by targeting of the endothelial ecto-ALP it decreases the adhesion of IRBCs to CD36. We performed a small randomized controlled trial in 21 patients with uncomplicated falciparum malaria on the antiadhesive effect of a single oral dose of 150 mg of levamisole as adjunctive treatment to quinine. It was shown that in patients treated with levamisole, significantly more late-stage parasites could be detected in the peripheral blood over time, which was attributed to the inhibition of sequestration with levamisole [17]. No adverse effects of levamisole were observed in this study. Vomiting is a common side effect of levamisole when used in repeated high doses as an adjunctive therapy with 5-fluorouracil in colon cancer chemotherapy, but is uncommon in low doses as used in antihelminth treatment and this study [18].

Because of these encouraging results, we performed an open-label randomized controlled trial of levamisole hydrochloride as adjuvant treatment in adult patients with severe falciparum malaria to study its effect on sequestration of infected red blood cells. At the time of the study, artesunate had replaced quinine as first-line treatment for severe malaria, so levamisole was studied as an adjunct to artesunate rather than quinine [19].

## METHODS

### Study Site and Patients

The study was conducted at Chittagong Medical College Hospital, Chittagong, Bangladesh, from May 2006 to August 2010. Chittagong Medical College Hospital is a 1000-bed teaching hospital with limited facilities for oxygen therapy, blood transfusion, intensive care, and renal dialysis. Malaria transmission is seasonal and of low intensity in this location. Ethical clearance was obtained from the Ministry of Health in Bangladesh, and from the Oxford Tropical Research Ethics Committee (registration number: ISRCTN 27232551).

Adult patients (aged ≥16 years) with slide-confirmed severe *P. falciparum* malaria (according to modified World Health Organization [WHO] criteria [20]) and parasitemia of >2% were recruited, provided that written informed consent was obtained from the patient or an attending relative.

Criteria for severe malaria included coma (Glasgow Coma Score [GCS] <11), pulmonary edema, repeated convulsions (≥2 in 24 hours), severe anemia (hematocrit level <20%, plus a parasite count >100 000 parasites/µL) or jaundice (bilirubin level >3.0 mg/dL, plus a parasite count >100 000 parasites/µL), renal failure (serum creatinine level >3 mg/dL), hypoglycemia (blood glucose level <40 mg/dL), shock (systolic blood pressure < 80 mm Hg with cool extremities), hyperparasitemia (peripheral asexual stage parasitemia >10%), hyperlactemia (venous plasma lactate >4 mmol/L), and/or acidemia (venous plasma bicarbonate level <15 mmol/L) [20].

Exclusion criteria were known allergies to levamisole or artesunate, >1 dose of another antimalarial treatment within 1 week before admission, pregnancy, and breastfeeding.

### Drug Treatment

Patients were randomly assigned to either adjunctive treatment with levamisole, or no adjunctive treatment, in addition to parenteral artesunate. Computerized randomization was balanced in blocks of 20, and allocation codes were kept in sealed envelopes. Patients, nurses, and laboratory scientists were masked to the treatment assigned. Levamisole treatment was directly observed by a study physician.

Levamisole was obtained as 25-mg tablets (Actavis Ltd, formerly Pharmamed Ltd) and was given simultaneously with the first dose of intravenous artesunate as a single dose of 150 mg (6 tablets of 25 mg) dissolved in sterile water. This dose is the same as that used in the treatment of *Ascaris lumbricoides* infections [18]. For all unconscious patients unable to swallow medication, a nasogastric tube was inserted. The dissolved levamisole was administered by this route. Those not allocated to receive levamisole were given only water. The solubility of levamisole is excellent (210 g/L). The recovery of levamisole hydrochloride aqueous solution from tablets has been reported to be 100% ± 2.1%, with good stability [21].

All patients were treated with parenteral artesunate (Guilin No. 2 Pharmaceutical Factory) according to the WHO treatment guidelines [19]. Supportive treatments were in accordance with WHO [19] and local hospital guidelines.

### Study Procedures

On admission, a full medical history and examination were carried out. Venous blood samples were obtained for hemoglobin, hematocrit, parasitemia, platelet count, white cell count, plasma lactate levels, glucose levels, and full biochemistry. Peripheral blood parasitemia and staging in thin films were assessed on admission from 2 separate blood samples, and then at 1, 2, 3, 4, 5, 6, 8, 10, and 12 hours after admission, followed by every 4 hours until parasite clearance, defined as 2 consecutive negative blood films.

### Assessment of Parasite Sequestration Dynamics

Primary outcome measures were dynamics of peripheral blood parasitemia and stage distribution [17]. These were assessed by measuring the area under the curve to 30 hours of the late stage–specific (late trophozoites and schizonts) parasite clearance curves comparing levamisole-treated patients with the control group.

In addition, the ratios between the observed and expected peripheral blood parasite densities of defined parasite developmental stages determined on the basis of validated morphological assessment were calculated at 6 and 12 hours after admission as sequestration ratios (SQRs) [17]. The SQR for a developmental stage of the parasite at a certain sample time point after admission was defined as follows:
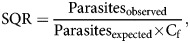

Where:

Parasites_observed_ is the observed number of parasites at the sample time, that is, the peripheral blood parasitemia per microliter of the developmental stage under consideration, assessed from a peripheral blood slide.

Parasites_expected_ is the expected number of parasites at the same sample time, that is, the number of parasites per microliter of that developmental stage expected to be present in the peripheral blood if the matching cohort of circulating young-form parasites on admission had developed unrestricted to the later developmental stage under consideration, without sequestration in the microvasculature, and without (splenic) clearance.

*C*_f_ is the fraction of parasites cleared by the spleen and through antimalarial drug action. This constant cannot be assessed in vivo, but was assumed to be the same in both treatment arms.

The area under the parasitemia-time curve over the first 30 hours for mature forms of the parasite (late trophozoites and schizonts) and the percentages of parasites that were trophozoites over the first 12 hours were also compared between the levamisole and control groups.

### Lactate

Plasma lactate was measured on admission, and then every 4 hours until parasite clearance, and then daily until full recovery of the patient. Lactate clearance time was the time to a decrease in plasma concentrations to <2 mmol/L (upper limit of normal value).

### Parasite Clearance

Parasite clearance rates and clearance slope half-lives were determined using the WorldWide Antimalarial Resistance Network Parasite Clearance Estimator [22]. Parasite clearance time was defined as the interval between start of treatment and the first of 2 sequential negative thick peripheral blood films. Parasite reduction ratios at 6 hours (PRR_6_), 12 hours (PRR_12_), 24 hours (PRR_24_), and 48 hours (PRR_48_) were defined as the ratio of the parasite count at admission to that at 6, 12, 24, or 48 hours [23].

### *Plasmodium falciparum* Histidine-Rich Protein 2

Plasma *P. falciparum* histidine-rich protein 2 (*Pf*HRP2) was assessed by enzyme-linked immunosorbent assay (Cellabs) from an EDTA plasma sample on admission. This provides an estimate of the total body parasite burden [24]. The total peripheral blood parasite burden was calculated from peripheral blood film parasite counts. The ratio of total to circulating parasite biomass as an indicator of the degree of sequestration was calculated by dividing these 2 parameters.

### Microvascular Flow

Microvascular blood flow was measured in capillaries in the rectal mucosa by orthogonal polarizing spectroscopy (OPS) [25] at 0, 6, and 24 hours. At each time point, 3 videos of minimum 10 seconds’ duration were recorded. These were then anonymized and scored by 2 independent blinded observers. In each video, 20 capillaries were scored as “flow,” “no flow,” or “uncertain,” and the proportion of capillaries with flow in the 3 videos per time point was calculated. The change in proportion of capillaries with flow over time was then used as the outcome measure.

### Levamisole Pharmacokinetics

A venous blood sample at 0, 4, 6, and 12 hours after levamisole administration was taken for pharmacokinetic assessment. Levamisole concentrations were determined using a high-performance liquid chromatography method adapted from Vandamme et al [26].

Plasma levamisole pharmacokinetics were characterized with nonlinear mixed-effects modeling using NONMEM version 6 (Icon Development Solutions). A first-order conditional estimation method allowing an interaction term was used for modeling, and basic diagnostics were visualized using Xpose 4.0 [27]. A one-compartment model with first-order absorption kinetics allowing a lag time has previously been described for levamisole in cancer patients [28]. This structural model was maintained as only sparse data were available from this study. An absorption lag time, however, was not used in this analysis as only one data point was collected during the absorption phase. Empirical Bayes estimates from the population model were used for individual predictions of maximum concentration and area under the plasma concentration-time curve (AUC). These pharmacokinetic parameters were correlated with the sequestration ratio as the primary pharmacodynamic measure of interest.

### Other Assessments

Fever was assessed at all sample times, as this can affect the cytoadherent properties of IRBCs [29]. Vital signs, oxygen saturation, level of consciousness (GCS), fluid input, and output were recorded every 6 hours until recovery. Patients were followed until discharge from the hospital.

### Statistical Procedures

Statistical analysis was performed using SPSS (version 15.0) and Stata software (version 10). Data were log-transformed to obtain a normal distribution, where appropriate. Normally distributed data were compared using Student *t* test, and the Mann–Whitney *U* test was used for nonpaired nonparametric data. Categorical data were compared by Pearson χ^2^ test and Fisher exact test, as appropriate. The Spearman rank test was used to assess correlations between nonnormally distributed data. Kaplan–Meier plots were constructed for time-dependent parameters. For comparison between treatment groups (Wilcoxon–Breslow test), the lactate clearance times in patients who died before clearance or recovery were set as maximum time (rather than censored), as persistent high plasma lactate concentrations are strongly associated with a fatal course of the disease [30]. The level of significance was set at *P* < .05.

The power calculation for this study was based on a previous in vivo study of uncomplicated falciparum malaria patients receiving levamisole plus quinine vs quinine alone [17]. A sample size of 60 patients was calculated, based on an expected standardized difference of 1 in the area under the time-parasitemia curve, a power of 85%, and a significance level of 5%.

## RESULTS

**Figure 1.**
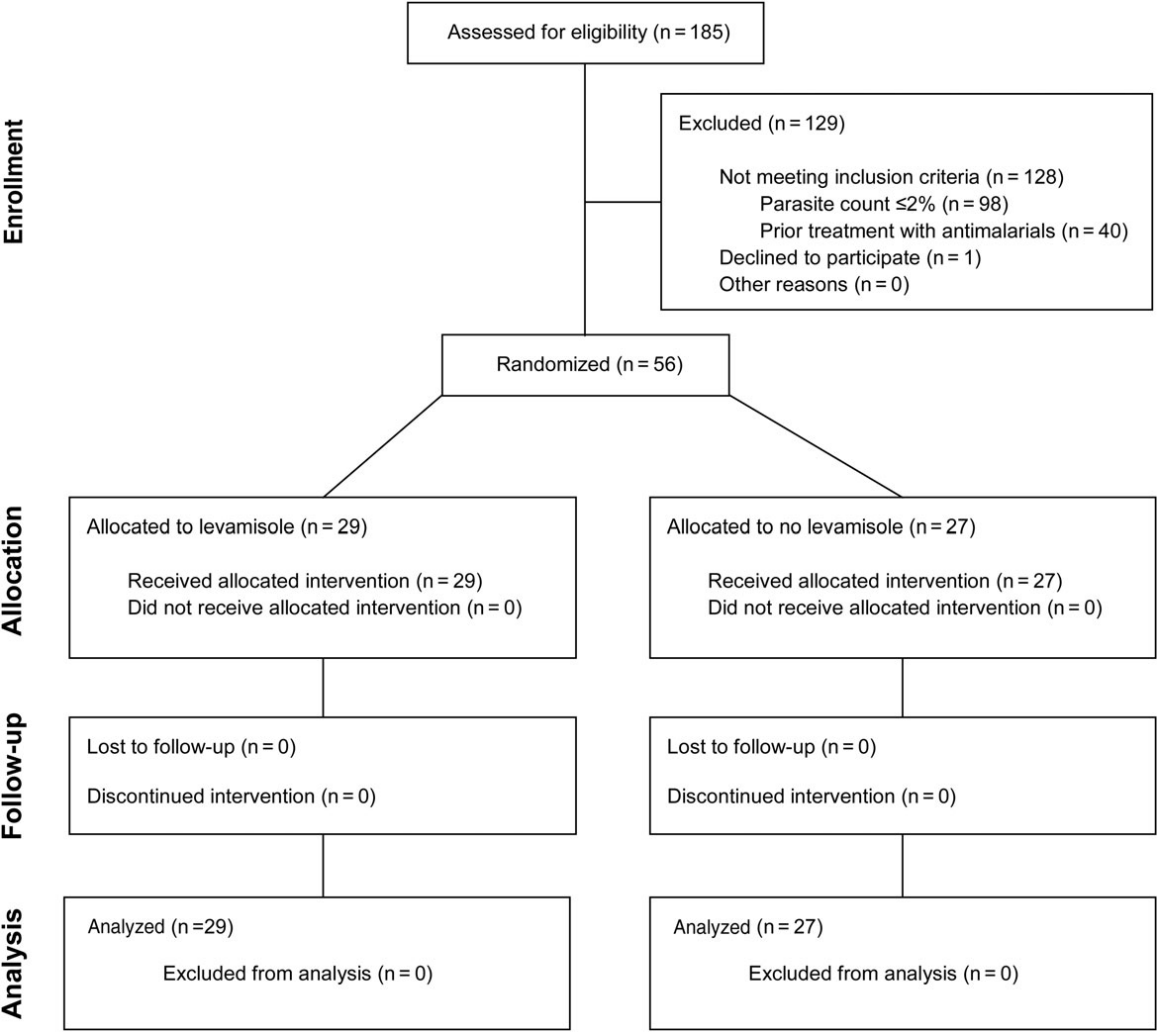
CONSORT (Consolidated Standards of Reporting Trials) diagram of the trial.

Fifty-six patients with severe malaria were enrolled and randomized to receive either levamisole as an adjunctive treatment to artesunate (n = 29) or artesunate alone (n = 27). A CONSORT diagram of the trial is shown in Figure 1.

### Baseline Characteristics

The baseline characteristics are summarized in Tables 1 and 2, and did not differ between treatment groups except for jaundice, which was slightly higher in the group not receiving levamisole. The plasma levels of *Pf*HRP2 and calculated total biomass are shown in Table 1. At baseline, there was no difference between the median ratio of sequestered to nonsequestered parasite biomass in those receiving levamisole (10.5 [95% confidence interval {CI}, 4.1–21.6]) and those not receiving levamisole (13.1 [95% CI, 8.8–35.6], *P* = .176).

**Table 1.** Baseline Characteristics

Variable	Levamisole (n = 29)	Control (n = 27)	*P* Value^a^
Age, y^b^	30 (25–45)	28 (21–45)	.774
Male/female sex, No.	18/11	22/5	.242
Temperature, °C	37.8 (37.4–38.3)	38.0 (37.5–38.6)	.589
Systolic blood pressure, mm Hg	104 (97–111)	105 (97–113)	.883
Oxygen saturation, %	95 (94–96)	95 (94–96)	.79
Glasgow Coma Score^b^	10 (8–15)	10 (6–13.5)	.167
Hematocrit, %	27.0 (23.3–30.7)	28.5 (25.2–31.7)	.942
Peripheral white blood cell count, cells/µL	10 559 (8510–12 608)	8504 (6946–10 062)	.329
Platelet count, cells/µL	94 630 (68 445–120 814)	72 960 (44 626–101 294)	.79
Parasitemia, parasites/µL^c^	294 775 (227 483–381 973)	236 742 (171 156–327 461)	.333
Trophozoites and schizonts, %^b^	3 (1–18)	3 (1–19)	.965
Plasma HRP2, µg/L	5802 (3336–8268)	6887 (3998–9777)	.578
Plasma HRP2-derived parasite burden, per patient^c^	1.09 × 10^13^ (5.95 × 10^12^–2.01 × 10^13^)	1.68 × 10^13^ (8.82 × 10^12^–3.25 × 10^13^)	.324
Ratio total/circulating parasite biomass	10.5 (4.1–21.6)	13.1 (8.8–35.6)	.176
Sequestered proportion of parasite biomass, %	85 (79–91)	90 (85–96)	.212
Serum creatinine, mg/dL^c^	1.5 (1.2–1.9)	1.6 (1.3–1.9)	.527
Total serum bilirubin, mg/dL^c^	3.3 (2.5–4.4)	4.2 (2.7–6.8)	.329
Serum albumin, g/100 mL	2.9 (2.6–3.2)	2.5 (2.3–2.8)	.083
Serum aspartate aminotransferase, U/L^c^	86 (67–110)	108 (82–141)	.412
Serum alanine aminotransferase, U/L^c^	24 (20–31)	33 (26–43)	.079
Plasma lactate, mmol/L^c^	4.6 (3.6–5.9)	5.4 (4.4–6.6)	.1
Venous bicarbonate, mmol/L	18.9 (16.9–20.9)	16.5 (14.7–18.3)	.126
Plasma base excess, mmol/L	−5.1 (−7.8 to −2.5)	−8.2 (−10.4 to −6.0)	.11

Data are presented as mean (95% confidence interval) unless otherwise indicated.

^a^ Student *t* test for normal distributed data and Mann–Whitney *U* test for the remainder.

^b^ Median (interquartile range).

^c^ Geometric mean (95% confidence interval).

**Table 2.** Markers of Severe Malaria Present on Admission

Marker	Levamisole (n = 29), No. (%)		Control (n = 27), No. (%)		*P* Value^a^
Glascow Coma Score <11	15	(52)	17	(63)	.396
Hematocrit <20% with parasite count >100 000 parasites/µL	4	(14)	3	(11)	1
Bilirubin >30 with parasite count >100 000 parasites/µL	0	(0)	4	(15)	.048
Serum creatinine >3 mg/dL	4	(14)	2	(7)	.671
Glucose <40 mg/dL	1	(3)	1	(4)	1
Shock (SBP <80 mm Hg plus cool peripheries)	2	(7)	1	(4)	1
Parasitemia >10%	15	(52)	10	(37)	.269
Venous lactate >4 mmol/L	13	(45)	15	(56)	.422
Venous bicarbonate <15 mmol/L	7	(24)	10	(37)	.251
Pulmonary edema	0	(0)	3	(11)	.106
Spontaneous bleeding	2	(7)	3	(11)	.664
Generalized convulsions (≥2 per 24 h)	7	(24)	4	(15)	.506

Abbreviation: SBP, systolic blood pressure.

^a^ χ^2^ test.

Five patients (17%) in the levamisole-treated group died, compared to 9 patients (33%) in the control group (*P* = .221). Median time to death was 1.21 (interquartile range [IQR], 0.97–1.5) hours for the levamisole group and 1.00 (IQR, 0.87–1.65) hours in the nonlevamisole group (*P* = .928). Of patients who died, 11 of 14 (79%) had cerebral malaria, 6 of 14 (43%) had severe acidosis, 1 of 14 (7%) had renal failure, and 3 of 14 (21%) developed acute respiratory distress syndrome. Postmortems were not possible in our study setting.

### Dynamics of Peripheral Blood Parasitemia and Stage Distribution

**Figure 2.**
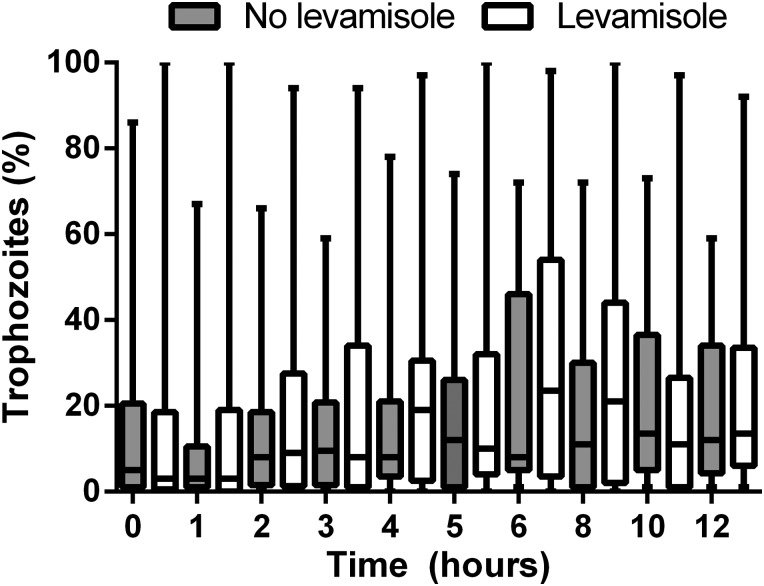
Percentage of trophozoites over time. Boxes represent median (quartiles) and whiskers represent range.

The median integrated number of late-stage parasites (late trophozoites and schizonts) in the peripheral blood slides during the first 30 hours after admission was 2150 (IQR, 0–28 025) parasites/µL × hour in the levamisole group compared to 5489 (IQR, 192–25 848) parasites/µL × hour in the control group (*P* = .25). The median integrated number of total trophozoites in peripheral blood slides during the first 30 hours after admission was 265 517 (IQR, 69 177–551 432) parasites/µL × hour in the levamisole group and 352 053 (IQR, 144 556–666 539) parasites/µL × hour in the control group (*P* = .515). The proportions of trophozoites in the peripheral blood slide over time are shown in Figure 2. There was no difference between treatment groups in the change over time in these proportions.

Table 3 shows the sequestration ratios compared between treatment groups. A higher sequestration ratio indicates prevention of sequestration if clearance (C_f_) is assumed to be similar in both groups. After 6 hours, the mean sequestration ratio for early trophozoites was >1 in both study arms, implying absence of significant sequestration or splenic clearance of early trophozoites at this time point. For all other stages at 6 and 12 hours, the sequestration ratio was <1, implying a high degree of sequestration or splenic clearance of the more mature parasite stages. There was no difference in the extent of sequestration measured as sequestration ratio between the 2 treatment arms.

**Table 3.** Sequestration Ratios

Time, Stage	Levamisole (n = 29)	Control (n = 27)	*P* Value^a^
SQR × C_f_ at 6 hours			
Early trophozoites (ET/LR)	1.28 (.57–2.91)	1.07 (.40–2.85)	.79
Late rings (LR/SR)	0.05 (.02–.13)	0.06 (.03–.12)	.70
Mid-trophozoites (MT/ET)	0.16 (.05–.54)	0.27 (.09–.85)	.54
SQR × C_f_ at 12 hours			
Early trophozoites (ET/SR)	0.05 (.02–.16)	0.10 (.04–.26)	.41
Small rings (SR/TR)	0.13 (.05–.36)	0.14 (.06–.35)	.91
Mid-trophozoites (MT/LR)	0.06 (.02–.23)	0.03 (.00–.16)	.47

Data are presented as geometric mean (95% confidence interval).

Abbreviations: C_f_, fraction cleared by the spleen, assumed the same for both treatment arms; ET, early trophozoites; LR, late rings; MT, mid-trophozoites; SQR, sequestration ratio; SR, small rings.

^a^ Student *t* test.

The parasite clearance rates, clearance slope half-lives, parasite clearance times, PRR_6_, PRR_12_, PRR_24_, and PRR_48_ are shown in Table 4. The PRR_6_, PRR_12_, PRR_24_, and PRR_48_ were greater in the levamisole group, but there was no difference in the other measures of parasite clearance. There was no difference in parasite clearance half-lives between fatal and nonfatal infections (median, 3.23 [IQR, 1.49–3.42] hours and 2.37 [IQR, 2.03–3.12] hours, respectively, *P* = .910).

**Table 4.** Parasite Clearance Measures

Clearance Measure	Levamisole (n = 29)	Control (n = 27)	*P* Value^a^
Clearance rate, per h	0.28 (0.26–0.31)	0.28 (0.25–0.32)	.791
Clearance half-life, h	2.5 (1.2–4.9)	2.5 (0.9–7.0)	.791
PRR_6_	2.2 (1.6–3.4)	1.2 (1.1–2.0)	.0015
PRR_12_	4.3 (2.5–5.2)	2.0 (1.5–3.8)	.029
PRR_24_	384.7 (61.3–1314.0)	11.8 (3.7–120.0)	.002
PRR_48_	9077.9 (6204.6–10987.7)	1272.6 (643.2–4469.0)	.037
PCT	48 (42–55)	48 (36–54)	.889

Data are presented as median (interquartile range).

Abbreviations: PRR_6_, PRR_12_, PRR_24_, and PRR_48_, parasite reduction ratio at 6, 12, 24, and 48 hours, respectively; PCT, parasite clearance time.

^a^ Mann–Whitney *U* test.

### Microvascular Flow

OPS was performed both on enrollment and at 6 and/or 24 hours in 12 patients in the levamisole group and 9 patients in the nonlevamisole group. Those who died before 6 or 24 hours could not have follow-up OPS, and OPS was not possible in those patients with significant diarrhea or who did not tolerate the procedure. There was no difference in improvement of microcirculatory flow assessed as the change in proportion of capillaries with flow: from 0 to 6 hours the median change was 6.7% (IQR, −10.5% to 26.8%) with levamisole vs 3.8% (−4.0% to 25.0%) without levamisole (*P* = .96); from 6 to 24 hours the median change was −0.7% (IQR, −5.0% to 14.2%) with levamisole vs 8.7% (IQR, −2.5% to 23.6%) without levamisole (*P* = .41); from 0 to 24 hours the median change was 16.8% (IQR, −0.75% to 26.7%) with levamisole vs 20.8% (−8.3% to 24.8%) without levamisole (*P* = .96). The median proportions of capillaries with flow in patients treated with levamisole were 77% (IQR, 68.5%–94%) at enrollment, vs 86.5% (IQR, 74.8%–99.0%) after 6 hours, and 91.5% (IQR, 86.8%–97.0%) after 24 hours; in patients not treated with levamisole, the median proportion was 78% (IQR, 64.8%–92.8%) at enrollment, 90.0% (IQR, 73.5%–95.0%) after 6 hours, and 91.5% (IQR, 68.8%–95.0%) after 24 hours, respectively.

### Lactate Clearance

**Figure 3.**
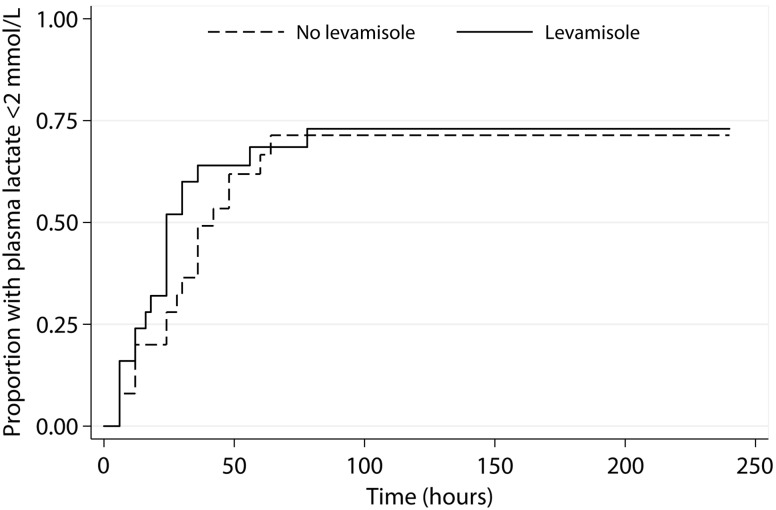
Kaplan–Meier plots of time to normalization of plasma lactate by treatment arm. *P* = .3.

Elevated admission plasma lactate concentrations (>2 mmol/L) were present in 48 of 56 patients (86%), 25 (86%) in the levamisole group and 23 (85%) in the nonlevamisole group. Baseline *Pf*HRP2 correlated with plasma lactate (Spearman *r* = 0.44, *P* = .001). Hyperlactemia (>4 mmol/L) was present in 13 of 29 (45%) and 15 of 27 (56%) patients, respectively. Median lactate clearance time was 24 hours (IQR, 12–30 hours) in the levamisole-treated and 28 (IQR, 12–36 hours) in the control group (*P* = .148, excluding patients who died). The Kaplan–Meier survival analysis for lactate clearance time (Figure 3) showed no difference between treatment groups (Wilcoxon–Breslow test, *P* = .3).

### Levamisole Pharmacokinetics

The mean maximum plasma concentration (C_max_) of levamisole for levamisole-treated patients was 703 (95% CI, 535–871) ng/mL, and the mean AUC was 10.7 (95% CI, 9.0–12.4) mg/L/hour. This is similar to values reported in previous studies [31]. There was no correlation between the sequestration ratio (at 6 and 12 hours) and levamisole C_max_ or AUC, except for the sequestration of early trophozoites at 6 hours (AUC *P* = .05, C_max_ *P* = .005) and small rings at 12 hours (AUC *P* = .03, C_max_ *P* = .6).

## DISCUSSION

In this study we were unable to detect a beneficial effect of levamisole as adjuvant therapy to intravenous artesunate in the treatment of adult severe malaria.

Our earlier study showed that sequestration was reduced by levamisole in patients with uncomplicated malaria treated with quinine, as indicated by an initial increase in peripheral late-stage parasitemia on peripheral blood smear, which was quantified by comparing sequestration ratios [17]. The present study shows that there is no significant change in sequestration in patients with severe malaria treated with levamisole as an adjuvant to intravenous artesunate.

The main difference with our earlier study is that in the current study, artesunate rather than quinine was used as antimalarial treatment. Quinine has a slow onset of action, and only kills trophozoite- and schizont-stage parasites [32]. In contrast, artesunate has a rapid onset of action within 4 hours after administration and a broad-stage specificity, also killing ring-stage parasites [32]. The pharmacodynamic properties of artesunate result in fast splenic clearance of ring-stage parasites, preventing their further maturation and sequestration in the microcirculation. Indeed, the appearance of late-stage parasites in the peripheral blood at 6 hours after admission or later was virtually absent in the current study, but prominent in uncomplicated malaria patients treated with quinine [17]. The calculated sequestration ratio, used to quantify sequestration, cannot distinguish between microcirculatory sequestration and splenic clearance. Splenic clearance is a minor confounder in the initial hours after slow-acting quinine, but a major confounder after rapidly acting artesunate [32]. The observed low sequestration ratios after start of treatment in the current study can thus express either high splenic clearance or microvascular sequestration. The greater parasite reduction ratio in patients treated with levamisole could be explained by a reduction in initial parasite sequestration, so that the parasites are exposed to splenic clearance. However, the parasite clearance rate after the initial lag time in clearance (shorter with levamisole) was the same in both treatment groups.

It is likely that the lack of effect of levamisole in the current study is the high parasiticidal potency of artesunate resulting in fast ring-stage and later-stage parasite clearance. Because this fast clearance by artesunate might have obscured the effect of levamisole, a beneficial effect as adjunctive treatment in severe malaria cannot be entirely excluded.

In addition, mechanisms of sequestration are thought to differ within the human host at different anatomical sites. In the peripheral endothelium, CD36 is primarily expressed, whereas in the brain ICAM and VCAM are more prominent. It is possible that in this study population of patients with severe malaria, endothelial receptors other than CD36 play a larger role mediating sequestration in the systemic circulation, thus reducing the potential effect of levamisole, which specifically inhibits cytoadherence to CD36.

Plasma lactate concentrations are a crude marker of tissue oxygenation and microcirculatory flow, and have a strong predictive value for mortality in severe malaria [30, 33]. In the current study, we could not show a difference between treatment groups in lactate clearance times or the Kaplan–Meier lactate survival curves. Again, a potential effect of levamisole might have been obscured by the potent action of artesunate.

Despite the presence of severe illness, absorption of levamisole was reliable with a mean plasma level of 96.5% ± 1.5 of that expected in healthy adults. In a previous study, the peak levels of levamisole in the plasma were reached after 1–2 hours [34]. The lack of a pharmacodynamic effect on sequestration can thus not be explained by inadequate absorption of levamisole after oral administration.

A variety of other adjunctive treatments aimed at reducing mortality from severe malaria have been evaluated in clinical trials without showing evidence of benefit, including adrenaline [35], anti–tumor necrosis factor antibody [36], aspirin [37], dexamethasone [38], heparin [37], human albumin [39], intravenous immunoglobulin [40], iron chelators [41, 42], low-molecular-weight dextran [43], mannitol [44, 45], N-acetylcysteine [46], phenobarbitone [47], and ursodeoxycholic acid [48].

In conclusion, we could not show a beneficial effect of levamisole as adjunctive therapy to artesunate in adult patients with severe falciparum malaria. It is possible that the potent and rapid onset of the parasiticidal effect of artesunate obscures the inhibiting effects on sequestration of levamisole.
